# *Mycobacterium tuberculosis* Infection among Asian Elephants in Captivity

**DOI:** 10.3201/eid2303.160726

**Published:** 2017-03

**Authors:** Gary Simpson, Ralph Zimmerman, Elena Shashkina, Liang Chen, Michael Richard, Carol M. Bradford, Gwen A. Dragoo, Rhonda L. Saiers, Charles A. Peloquin, Charles L. Daley, Paul Planet, Apurva Narachenia, Barun Mathema, Barry N. Kreiswirth

**Affiliations:** Albuquerque Biopark, Albuquerque, New Mexico, USA (G. Simpson, R. Zimmerman, M. Richard, C.M. Bradford, G.A. Dragoo, R.L. Saiers);; Rutgers University Public Health Research Institute, Newark, New Jersey, USA (E. Shashkina, L. Chen, B.N. Kreiswirth);; University of Florida College of Pharmacy, Gainesville, Florida, USA (C.A. Peloquin);; National Jewish Health, Denver, Colorado, USA (C.L. Daley);; Children’s Hospital of Philadelphia, Philadelphia, Pennsylvania, USA (P. Planet);; American Museum of Natural History Sackler Institute for Comparative Genomics, New York, New York, USA (A. Narachenia);; Columbia University Mailman School of Public Health, New York (B. Mathema)

**Keywords:** Mycobacterium tuberculosis, tuberculosis and other mycobacteria, bacteria, Asian elephants, *Elephas maximus*, transmission, whole-genome sequencing, bacterial infection, zoonoses

## Abstract

Although awareness of tuberculosis among captive elephants is increasing, antituberculosis therapy for these animals is not standardized. We describe *Mycobacterium tuberculosis* transmission between captive elephants based on whole genome analysis and report a successful combination treatment. Infection control protocols and careful monitoring of treatment of captive elephants with tuberculosis are warranted.

Over the past 20 years, recognition of infection and disease caused by *Mycobacterium tuberculosis* in captive elephants and their keepers in the United States and globally has grown (*1*). We describe the diagnosis and treatment of 2 cases of active tuberculosis (TB), separated by 12 years, in 2 Asian elephants in a closed, captive population. In addition, we describe molecular and comparative genomic analysis of *M. tuberculosis* strains cultured from each elephant to investigate transmission.

## The Study

In July 1997, the city of Albuquerque, New Mexico, USA, acquired 2 elephants that had been subject to poor conditions in a small traveling circus. Elephant A, a 31-year-old Asian elephant, and elephant B, an 8-year-old African elephant, were quarantined together in an isolated section of the Albuquerque Biopark modified to hold elephants. Quarterly trunk washings (*2*) taken from both elephants over the course of 1 year of quarantine were negative for *M. tuberculosis* by culture. Both elephants were subsequently transferred to the zoo in late 1998.

**Figure 1:**
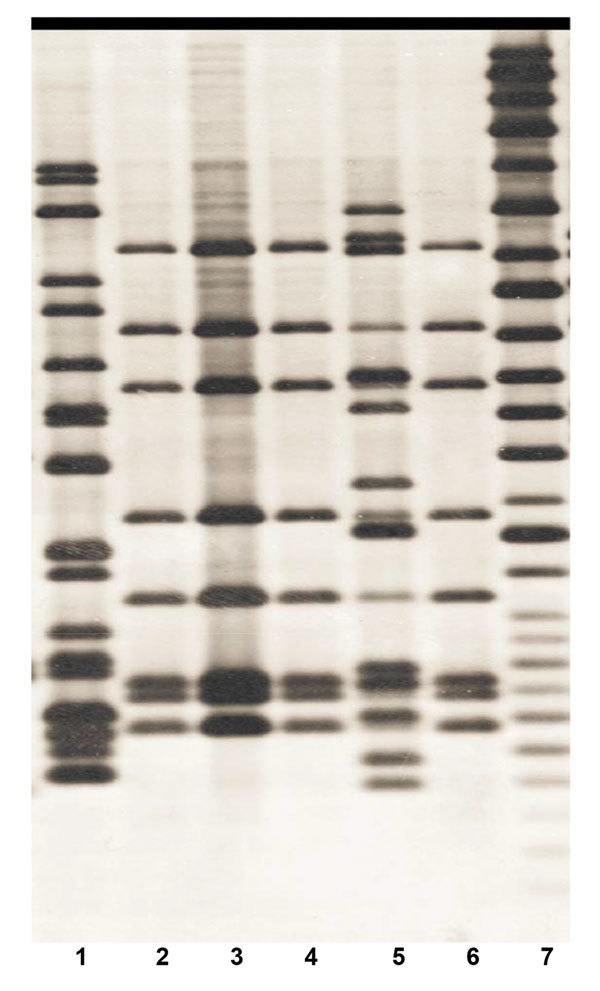
IS*6110* Southern blot hybridization patterns of 6 *Mycobacterium tuberculosis* isolates recovered from elephants A (lanes 1–5) and C (lane 6) (*4*) in study of tuberculosis in captive elephants, Albuquerque, New Mexico, USA, 1997–2013. The fingerprint pattern in lane 1 types the strain to principal genetic group 1, the fingerprint pattern in lanes 2–4 and lane 6 types the strain to principal genetic group 2 and the fingerprint pattern in lane 5 types the strain to principal genetic group 3. Lane 7, molecular mass standard.

Trunk washings taken from the zoo’s elephant herd every 6 months were negative for *M. tuberculosis* by culture until October 2000, when a specimen from elephant A was found to be positive on 7H11 bi-plates. Elephant A had TB diagnosed and was isolated and monitored, having 3 trunk washings collected over each 7-day period; this cycle continued until week 12 of treatment, when all 3 washings from that week were negative for *M. tuberculosis*. Among the 13 cultured *M. tuberculosis* isolates recovered from 6 different collections, IS*6110* genetic analysis (*3*,*4*) identified 3 different strains (Figure 1). Antimycobacterial susceptibility testing (*5*) revealed that 1 strain was mono-rifampin resistant and all others were pansusceptible.

**Figure 2:**
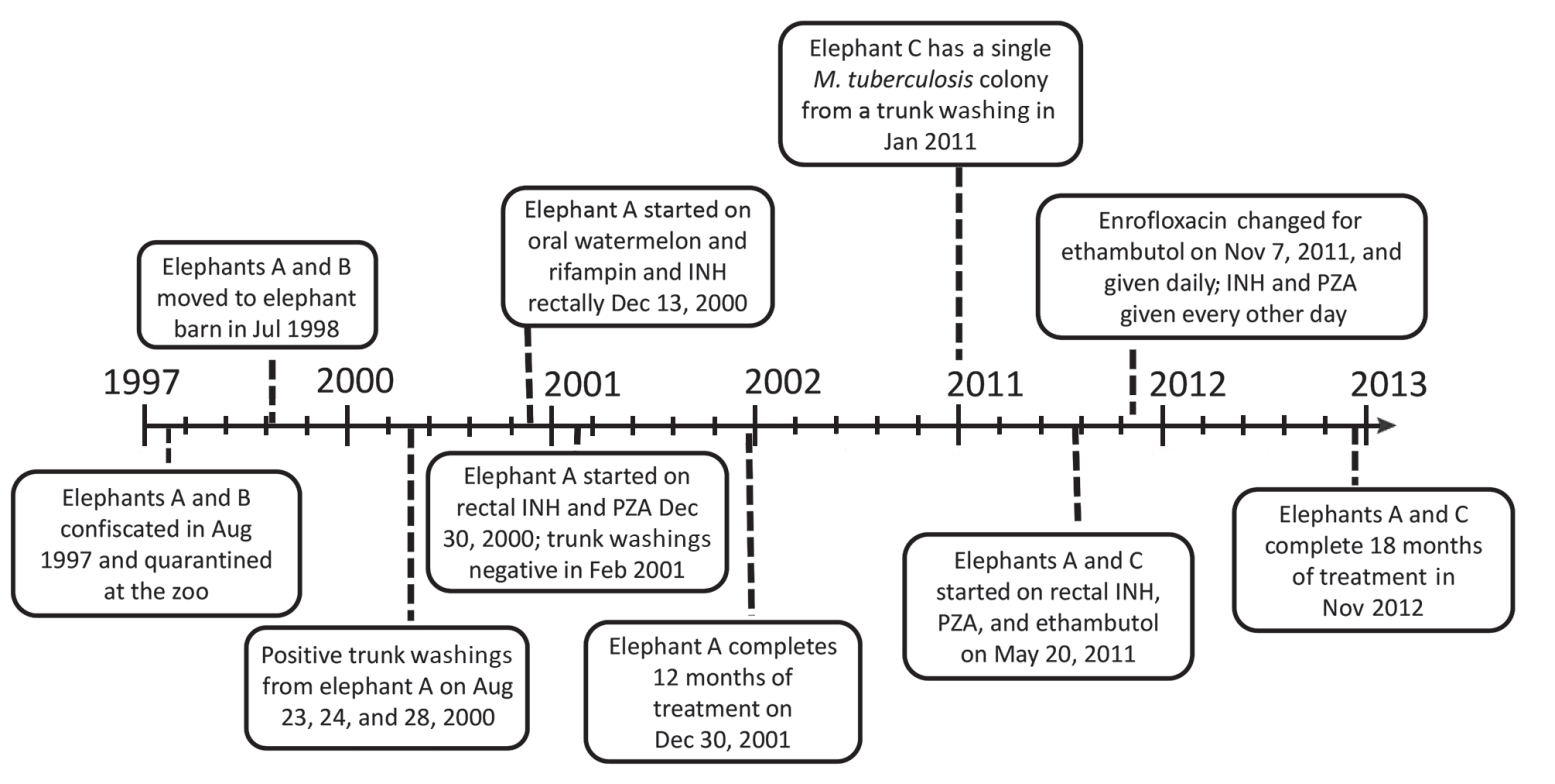
Annotated timeline documenting the course of events in study of tuberculosis in captive elephants, Albuquerque, New Mexico, USA, 1997–2013. INH, isoniazid; PZA pyrazinamide.

Initial treatment efforts included an anti-TB regimen of isoniazid (5 mg/kg), rifampin (10mg/kg), and pyrazinamide (25 mg/kg). This regimen was initially given orally, but the administration failed because of elephant’s A refusal to ingest the medication despite attempts to disguise or mix the drugs with treats or other food. As a result, isoniazid and pyrazinamide were given rectally, with serum concentrations obtained at 1, 2, and 4 hours after administration. This regimen was continued daily for 2 months, then every other day (QOD) for 1 year (Figure 2). The lengthy 3-month period until trunk washings were negative for *M. tuberculosis* by culture, the use of only 2 drugs, and the switch to a QOD regimen after only 2 months raised concern that elephant A’s treatment was suboptimal. In particular, pyrazinamide attacks a specific subpopulation of organisms, and in humans, the selection of drug resistance to other agents in the treatment regimen is increased (*6*).

In response to elephant A’s *M. tuberculosis*–positive trunk washings and contact history, elephant B was segregated from the herd and given isoniazid rectally QOD for 6 months as preventive therapy. Both elephants tolerated daily isoniazid poorly, having observable depression and loss of appetite. Clinical signs improved substantially with the start of QOD dosing, and both elephants completed therapy at the end of December 2001.

In December 2010, an Asian elephant (elephant C) had an *M. tuberculosis*–positive serologic test result by Chembio DPP VetTB Assay for Elephant (Chembio Diagnostic Systems, Inc., Medford, NY, USA) (*7*). An aggressive trunk washing testing cycle of 3 consecutive days on, followed by 3 days off, was initiated. A specimen from January 2011 produced a single colony of *M. tuberculosis* on 7H11 agar. Genetic analysis revealed an identical IS*6110* fingerprint pattern (Figure 1, lane 6) to the predominant pansusceptible strains isolated from elephant A (Figure 1, lanes 2, 3, and 4). Elephant C’s isolate was pansusceptible to all first-line anti-TB drugs. Treatment of elephant C was initiated in May 2011 by published guidelines (*2*); in this case, ethambutol was added to isoniazid and pyrazinamide with the same dosing used to treat elephant A. Routine therapeutic monitoring of serum concentrations led to discontinuing ethambutol and instituting enrofloxacin, a 4-fluoroquinolone used primarily in veterinary settings (*6*). Treatment was completed after 18 months. The previous concern that elephant A had received suboptimal therapy led to her retreatment in concert with elephant C.

A contact investigation was performed among the entire Albuquerque Biopark staff (178 persons) by using tuberculin skin testing and an interferon-gamma release assay (*8*,*9*). No evidence of *M. tuberculosis* transmission was found.

The *M. tuberculosis* isolate recovered from elephant C exhibited an identical IS*6110* fingerprint pattern to the EH3 isolate from elephant A (Figure 1). Although transmission between elephants A and C is not certain, exposure occurred before elephant’s A first positive trunk washing, and no coincident TB disease was detected among the Albuquerque Biopark staff. More than 10 years after cohabitation with elephant A in the elephant barn, elephant C had onset of active TB caused by an isolate that had the identical IS*6110* fingerprint to the infecting *M. tuberculosis* isolate from elephant A. Comparative whole genome sequencing analysis of the EH3 *M. tuberculosis* strains from elephants A and C was performed by using an Illumina MiSeq platform (Illumina, Inc., San Diego, CA, USA) (*10*). Single-nucleotide polymorphism (SNP) analysis revealed a total of 3 nt changes (compared with the reference genome H37Rv [GenBank accession no. NC_000962]) at positions 10774 (C to T), 2492143 (C to G), and 3013272 (T to C) during the 11-year period, a rate consistent with the modeled 0.3 SNPs per genome per year as previously reported (*10*,*11*). The sequencing data has been deposited in the National Center for Biotechnology Information database under the BioProject ID no. PRJNA328788.

Recently, whole genome sequencing was used to analyze genetic variation among strains from relapsed patients with recurrent TB (*10*) and to establish genomic mutation rates in a study with nonhuman primates (*11*). Remarkably, both studies showed limited genomic variation and yielded the consensus that *M. tuberculosis* chromosomes change at a rate 0.3 SNPs per year.

## Conclusions

Our whole-genome sequencing analysis confirmed the molecular identity of 2 *M. tuberculosis* isolates recovered 12 years apart from 2 captive elephants. The remarkable conservation between the 2 genomes is consistent with previous studies (*10**,**11*). Because elephant C had routine trunk washings that were negative during this period and no intermittent shedding occurred, we believe elephant C had latent TB and that elephant A was the source of the infection during their cohabitation. In support of the probable transmission between elephants A and C, the records at the Albuquerque Biopark confirm that both elephants were barned at the end of the each day from July 1998 through August 2000, when elephant A had a positive trunk washing and was isolated.

Numerous challenges were met in managing the treatment and control of TB among the elephants in the Biopark, including the choice of antimycobacterial agents, their comprehensive delivery, and the appropriate length of treatment. Rectal administration proved the most efficacious, and this approach was adopted for treating elephants A and C and for preventive treatment of elephant B. The availability of enrofloxacin, a fluoroquinolone not used in human health, appeared to be an effective third agent to combine with isoniazid and pyrazinamide, although prospective study data are lacking. This $20,000 regimen (per elephant per year) was ultimately used for 1 year to successfully treat elephant C and to retreat elephant A. The fact that no skin test conversions or TB disease were documented among Biopark staff with epidemiologic association to the elephants supports the evidence that transmission did not involve humans at that location.
