# Correction: Vol. 23, No. 3

**DOI:** 10.3201/eid2304.C12304

**Published:** 2017-04

**Keywords:** errata, erratum, correction

The name of author Apurva Narechania was misspelled in *Mycobacterium tuberculosis* Infection among Asian Elephants in Captivity (G. Simpson et al.). The article has been corrected online (https://wwwnc.cdc.gov/eid/article/23/3/16-0726_article).

